# Blood urea nitrogen-to-albumin ratio as a new prognostic indicator of 1-year all-cause mortality in patients with IPF

**DOI:** 10.3389/fmed.2024.1497530

**Published:** 2025-01-06

**Authors:** Shaobo Ge, Yuer Li, Rui Li, Jin Liu, Rui Zhang, Hongyan Fu, Jingjing Tang, Jie Zhang, Nali Zhang, Ming Zhang

**Affiliations:** ^1^Department of Respiratory and Critical Care Medicine, The Second Affiliated Hospital of Xi’an Jiaotong University, Xi’an, China; ^2^Department of Respiratory and Critical Care Medicine, Xi’an No.3 Hospital, The Affiliated Hospital of Northwest University, Xi’an, China; ^3^Department of Respiratory and Critical Care Medicine, Luoyang Hospital, The Second Affiliated Hospital of Xi’an Jiaotong University, Luoyang, China

**Keywords:** idiopathic pulmonary fibrosis, prognosis, urea nitrogen, albumin, biomarker

## Abstract

**Background:**

Idiopathic pulmonary fibrosis (IPF) is an interstitial lung disease characterized by chronic inflammation and progressive fibrosis. The blood urea nitrogen-to-albumin ratio (BAR) is a comprehensive parameter associated with inflammation status; however, it is unknown whether the BAR can predict the prognosis of IPF.

**Methods:**

This retrospective study included 176 patients with IPF, and 1-year all-cause mortality of these patients was recorded. A receiver operating characteristic (ROC) curve was used to explore the diagnostic value of BAR for 1-year all-cause mortality in IPF patients, and the survival rate was further estimated using the Kaplan–Meier survival curve. Cox proportional hazards regression model and forest plot were used to assess the association between the BAR and 1-year all-cause mortality in IPF patients.

**Results:**

The BAR of IPF patients was significantly higher in the non-survivor group than in the survivor group [0.16 (0.13–0.23) vs. 0.12 (0.09–0.17) mmol/g, *p* = 0.002]. The area under the ROC curve for predicting 1-year all-cause mortality in IPF patients was 0.671, and the optimal cut-off value was 0.12 mmol/g. The Kaplan–Meier survival curve showed that the 1-year cumulative survival rate of IPF patients with a BAR ≥0.12 was significantly decreased compared with the patients with a BAR <0.12. The Cox regression model and forest plot showed that the BAR was an independent prognostic biomarker for 1-year all-cause mortality in IPF patients (HR = 2.778, 95% CI 1.020–7.563, *p* = 0.046).

**Conclusion:**

The BAR is a significant predictor of 1-year all-cause mortality of IPF patients, and high BAR values may indicate poor clinical outcomes.

## Introduction

Idiopathic pulmonary fibrosis (IPF) is a serious lung condition characterized by progressive respiratory distress and decreased pulmonary function. However, the etiology and pathogenesis of IPF remain largely unknown (1). The global incidence of IPF is estimated to vary between 1 and 13 cases per 100,000 individuals, and its prevalence may range from 3 to 45 cases per 100,000 individuals (2). The clinical prognosis of IPF is poor, with a median survival period estimated to be only 3–5 years after the initial diagnosis (2). Therefore, it is very important to identify IPF patients with a high mortality risk, so that early appropriate treatment can improve their prognosis.

Several biomarkers can be used to predict the clinical outcomes of IPF patients. For example, it has been reported that a 6-min walk test, pulmonary function parameters, and partial pressure of oxygen in the arterial blood (PaO_2_) are associated with IPF prognosis (3–5). Another study has demonstrated that the serum levels of matrix metalloproteinases-7, intracellular adhesion molecule-1, and interleukin-8 are potential prognostic indicators for IPF (6), while all of the above biomarkers have not been widely used to predict the prognosis of IPF patients in clinical practice due to the reliability, economic efficiency, and accessibility of these biomarkers. Hence, there is significant clinical value in exploring an accessible, cost-effective, and non-invasive blood biomarker to assess the risk of mortality in IPF patients.

Blood urea nitrogen (BUN) is a major product of protein metabolism in the human body, and its levels can increase with renal dysfunction and malnutrition. Albumin is one of the most commonly used assays in patients with IPF (7), and decreased serum albumin may reflect ongoing inflammation, malnutrition, or loss of albumin’s protective effects (inhibition of endothelial cell apoptosis, antioxidant effects, and reduced platelet aggregation) (8). The blood urea nitrogen-to-albumin ratio (BAR) is a comprehensive parameter that reflects the inflammatory and nutritional status and has been used as a prognostic indicator for several diseases. For example, it has been demonstrated that the BAR is a reliable predictor for all-cause mortality in patients with chronic obstructive pulmonary disease (COPD) (9) and hepatitis B viral cirrhosis (10). Another study has found that the BAR has a significant relationship with the time to mortality in community-acquired pneumonia (11). It is well-known that IPF is an inflammatory disease usually associated with malnutrition, but it is still unclear whether the BAR can be used as a biomarker to predict the outcomes of IPF patients. Therefore, the objective of this study was to evaluate the prognostic value of BAR levels for 1-year all-cause mortality in patients with IPF.

## Methods

### Subjects

**Figure 1:**
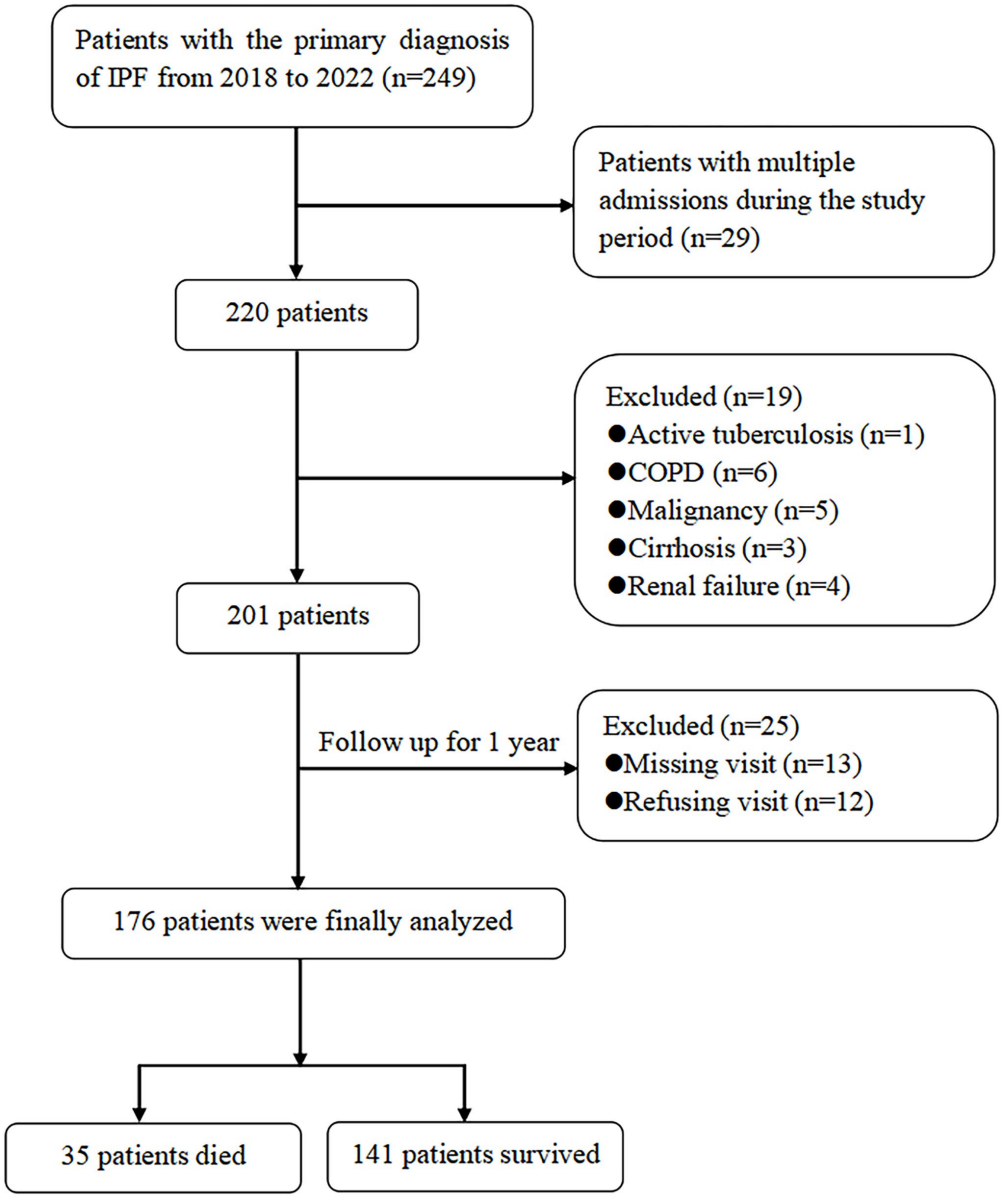
Flowchart of study patients.

A total of 249 patients with IPF who received medical treatment at the Department of Respiratory and Critical Care Medicine, the Second Affiliated Hospital of Xi’an Jiaotong University, from January 2018 to July 2022, were retrospectively analyzed. The diagnostic criteria for IPF are based on the ATS/ERS/JRS/ALAT clinical practice guidelines for IPF (1, 12), which depend on the identification of the usual interstitial pneumonia (UIP) pattern on high-resolution computed tomography (HRCT) and the exclusion of other causes of pulmonary fibrosis, such as occupational or environmental exposures and autoimmune connective tissue diseases. Autoimmune connective tissue diseases were excluded by clinical manifestations and laboratory tests, such as autoantibodies, rheumatoid factor, and anti-neutrophil cytoplasmic antibodies. The inclusion criteria required a definite diagnosis of IPF and an age of 40 years or older. For patients with multiple hospitalizations during the study period, only the first visit was included. IPF patients were also excluded from the present study if they had a combination of active tuberculosis, COPD, malignancy, cirrhosis, or renal failure. Follow-up data were obtained by inpatient visits and telephone calls for 1 year from the date of admission. Finally, 176 patients were included in the present study, and 35 patients died during the 12-month follow-up (Figure 1). This study was approved by the Research Committee of Human Investigation of the Second Affiliated Hospital of Xi’an Jiaotong University, and all patients gave informed consent.

### Treatment of IPF

The primary treatment objectives for hospitalized IPF patients were to improve symptoms and quality of life. Some patients received methylprednisolone via intravenous infusion, which was tapered off as their symptoms improved. Antibiotics were administered if bacterial infection was suspected and adjusted according to clinical symptoms and signs, sputum culture tests, and biochemical inflammatory markers. Oxygen was provided to maintain oxygen saturation ≥ 90%. Antifibrotic therapy with pirfenidone or nintedanib was recommended for all hospitalized patients, but some of them were reluctant to use antifibrotic drugs.

### Clinical and biochemical examinations

The basic clinical information of all participants was recorded in detail. Smoking history, medical history, and information about the use of antifibrotic medication were also collected. Fasting venous blood samples of all patients were collected at the beginning of hospitalization, and routine blood tests, liver function, and renal function were determined in the clinical laboratory.

### Blood gas analysis and pulmonary function

PaO_2_, partial pressure of carbon dioxide (PaCO_2_) in the arterial blood, and pH were immediately measured using a blood gas analyzer (ABL90 FLEX, Radiometer, Denmark) on the first day of hospitalization. The oxygenation index (OI) was further calculated as the ratio of PaO_2_ to the fraction of inspired oxygen. All enrolled patients underwent pulmonary function tests using spirometry (GANSHORN, Germany) either within 1 week prior to hospitalization or during their hospital stay.

### The gender-age-physiology index

The GAP index included gender, age, forced vital capacity (FVC), and diffusing capacity of carbon monoxide (DLco), which has been widely used to evaluate the severity of IPF patients (13). It is calculated as follows (13): 1 point for men, age 61–65 years, FVC 50–75%, or DL_CO_ 36–55%; 2 points for age > 65 years, FVC < 50%, or DL_CO_ ≤ 35%; and 3 points for inability to perform spirometry.

### Statistical analysis

All quantitative data were examined using the Kolmogorov–Smirnov test for normal distribution, and they are expressed as mean ± standard deviation (SD) or median (interquartile range) depending on the distribution status. Categorical variables are presented as percentages. Differences between the two groups were determined using Student’s *t*-test, the Mann–Whitney U-test, or the chi-square test. The receiver operating characteristic (ROC) curve was used to determine the BAR threshold for 1-year all-cause mortality in patients with IPF, and the diagnostic efficacies of the BAR and the GAP index were compared using the DeLong test. Spearman’s method was applied to explore the correlation between the BAR value and other parameters, such as OI, FVC % predicted, and DL_CO_ % predicted. The survival curve was drawn using the Kaplan–Meier method, and cumulative survival rates were analyzed using the log-rank test. Variables detected in the univariate Cox analyses with a *p*-value less than 0.05 were included in the multivariate COX regression analysis to illustrate the risk of mortality in IPF, and a forest plot was also plotted. Statistical analyses were conducted using SPSS version 27.0 software (SPSS Inc., Chicago, IL, United States), and a *p*-value of less than 0.05 was considered statistically significant.

## Results

### Baseline characteristics of the study population

The baseline clinical characteristics of the IPF patients are presented in Table 1, and 176 patients with available follow-up survival data were finally included. The parameters of age, sex, and smoking index, along with the coexistence rate of hypertension or diabetes, were not significantly different between the survivors and non-survivors (all *p* > 0.05). Non-survivors had a higher coexistence rate of coronary heart disease and a lower percentage of antifibrotic medication than the survivors (22.9% vs. 7.1% and 68.6% vs. 94.3%, respectively, both *p* < 0.05). Compared to the survivor group, pulmonary parameters (FVC and DL_CO_) significantly decreased, and the GAP index notably increased in the non-survivor group (all *p* < 0.001). The BAR value was significantly increased in the non-survivors compared to the survivors [0.16 (0.13–0.23) vs. 0.12 (0.09–0.17) mmol/g, *p* = 0.002]. There were also significant differences in albumin and BUN between the two groups (both *p* < 0.05). We also found that the body mass index (BMI), PaO_2_, and OI significantly decreased in the non-survivor group compared to the survivor group (*p* < 0.05). Furthermore, the levels of alanine aminotransferase, cystatin C, and leukocyte count were all significantly higher in the non-survivor group than those in the survivor group (*p* < 0.05). However, there were no significant differences in pH, PaCO_2_, direct bilirubin, indirect bilirubin, aspartate aminotransferase, globulin, and hemoglobin between the survivors and non-survivors (*p* > 0.05).

**Table 1:** Clinical and physiological characteristics of the study population.

Characteristic	Total	Survivors	Non-survivors	*p*- value
Number	176	141	35	
Age (yr.)	67.03 ± 9.96	67.01 ± 9.97	67.11 ± 10.05	0.958
Male subjects (%)	73.3	70.2	85.7	0.060
BMI (kg/m^2^)	23.19 ± 2.73	23.49 ± 2.71	21.96 ± 2.49	0.003
Smoking index (pack-year)	1.03 (0.00–30.00)	0.00 (0.00–30.00)	15.00 (0.00–20.00)	0.670
Smoking status				
Never (%)	48.8	51.1	40.0	
Current (%)	13.1	15.6	2.9	
Ever smoked (%)	38.1	33.3	57.1	
Comorbidity				
Hypertension (%)	30.6	29.8	34.3	0.600
Diabetes (%)	21.0	22.0	17.1	0.530
Coronary heart disease (%)	10.2	7.1	22.9	0.020
Antifibrotic medication (pirfenidone or nintedanib, %)	89.2	94.3	68.6	0.000
pH	7.43 ± 0.33	7.42 ± 0.27	7.44 ± 0.51	0.189
PaCO_2_ (mmHg)	37.81 ± 5.89	38.10 ± 3.93	36.63 ± 10.65	0.426
PaO_2_ (mmHg)	74.26 ± 13.00	76.05 ± 11.73	67.03 ± 15.37	0.000
OI (mmHg)	336.24 ± 79.33	355.83 ± 57.22	257.37 ± 104.41	0.000
FVC (% predicted)	74.27 ± 18.38	77.49 ± 15.86	61.37 ± 22.06	0.000
DL_CO_ (% predicted)	70.17 ± 22.51	72.95 ± 23.51	59.04 ± 13.20	0.000
GAP index	3.00 (2.00–4.00)	3.00 (2.00–4.00)	4.00 (3.00–5.00)	0.000
DBIL (μmol/L)	3.02 (2.34–4.13)	3.00 (2.29–3.97)	3.08 (2.59–4.73)	0.158
IBIL (μmol/L)	9.43 (6.61–12.69)	9.38 (6.60–12.66)	10.08 (6.70–13.20)	0.505
ALT (IU/L)	15.00 (11.00–25.00)	15.00 (11.00–23.00)	19.00 (13.90–34.00)	0.020
AST (IU/L)	21.00 (18.00–25.00)	20.00 (17.05–24.00)	23.00 (18.00–34.00)	0.052
Albumin (g/L)	36.09 ± 4.72	36.58 ± 4.50	34.12 ± 5.12	0.005
Globulin (g/L)	29.62 ± 5.86	29.38 ± 5.87	30.58 ± 5.82	0.278
BUN (mmol/L)	5.11 ± 1.87	4.90 ± 1.68	5.98 ± 2.35	0.014
Creatinine (μmol/L)	58.66 ± 15.59	57.65 ± 15.65	62.70 ± 14.87	0.086
eGFR (ml/min/1.73m^2^)	100.21 ± 15.11	100.08 ± 15.29	100.71 ± 14.56	0.826
Cystatin C (mg/L)	1.05 ± 0.24	1.01 ± 0.22	1.17 ± 0.30	0.007
BAR (mmol/g)	0.13 (0.10–0.17)	0.12 (0.09–0.17)	0.16 (0.13–0.23)	0.002
Leukocyte count (×10^9^/L)	6.99 (5.80–8.50)	6.71 (5.67–8.20)	8.01 (6.30–11.58)	0.006
HGB (g/L)	137.51 ± 19.53	137.50 ± 19.50	137.54 ± 20.05	0.990
Platelet count (×10^9^/L)	206.01 ± 73.83	203.37 ± 70.60	216.66 ± 85.96	0.342

Data are expressed as mean ± standard deviation or median (interquartile range) or percentage. BMI, body mass index; PaCO_2_, partial pressure of carbon dioxide in arterial blood; PaO_2_, partial pressure of oxygen in arterial blood; OI, oxygenation index; FVC, forced vital capacity; DL_CO_, diffusion capacity of carbon monoxide; GAP, gender-age-physiology; DBIL, direct bilirubin; IBIL, indirect bilirubin; ALT, alanine aminotransferase; AST, aspartate aminotransferase; BUN, blood urea nitrogen; eGFR, estimated glomerular filtration rate; BAR, blood urea nitrogen-to-albumin ratio; HGB, hemoglobin.

### Correlations of BAR with OI, FVC % predicted, and DL_CO_ % predicted

**Figure 2:**
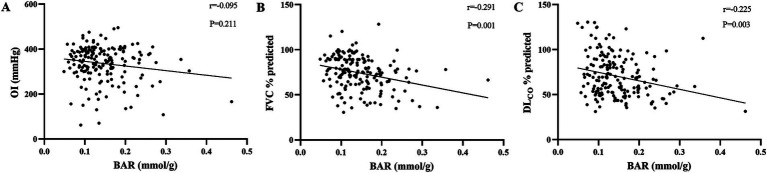
Correlations of the BAR with well-known prognostic biomarkers of IPF. Correlations of the BAR with OI **(A)**, FVC % predicted **(B)**, and DL_CO_ % predicted **(C)** were analyzed using Spearman’s test. BAR, blood urea nitrogen-to-albumin ratio; OI, oxygenation index; FVC, forced vital capacity; DL_CO_, diffusion capacity of carbon monoxide.

The correlations between the BAR and other parameters in IPF were studied using Spearman’s analysis, and the results are presented in Figure 2. We found that the BAR was significantly negatively correlated with FVC % predicted and DL_CO_ % predicted (*r* = −0.291 *p* = 0.001 and *r* = −0.225 *p* = 0.003, respectively), while there was no significant correlation between the BAR and OI (*r* = −0.095, *p* = 0.211).

### Predictive value of BAR for 1-year all-cause mortality in IPF patients

**Figure 3:**
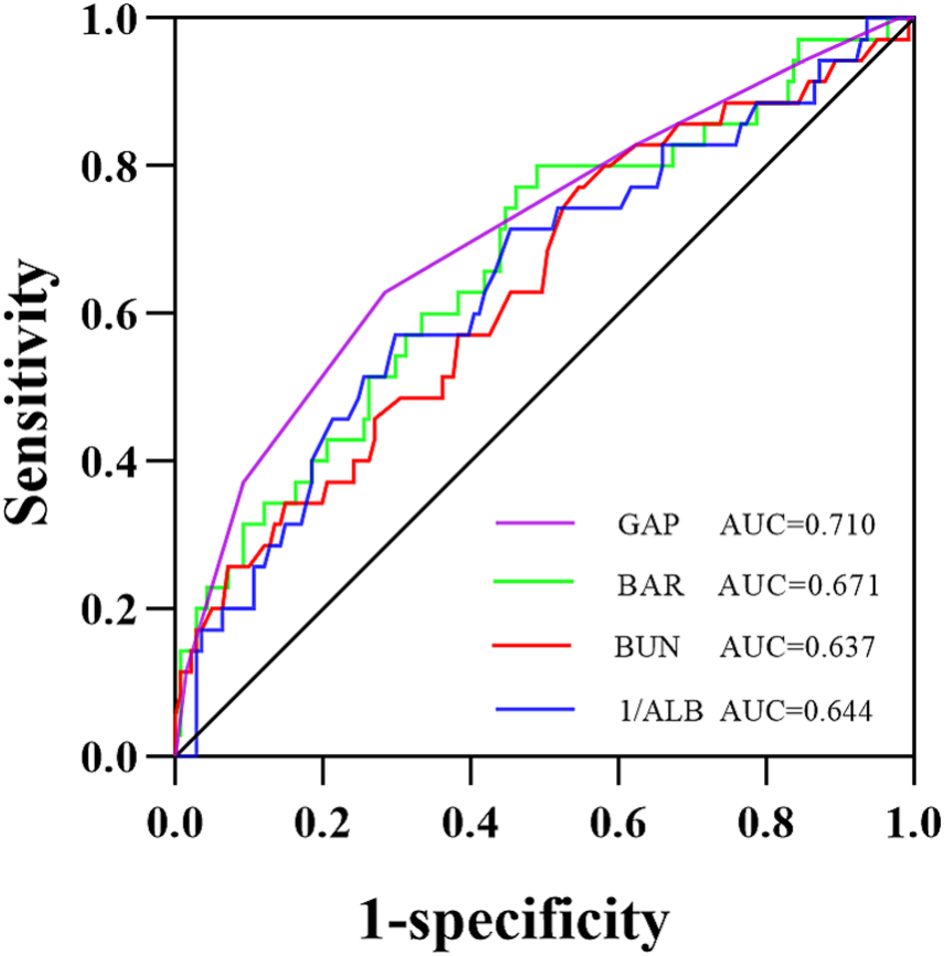
ROC curve analysis for predicting 1-year all-cause mortality in IPF patients. The area under the ROC curve was 0.671 for the BAR, 0.637 for BUN, 0.644 for 1/ALB, and 0.710 for the GAP index. ROC, receiver operating characteristic; AUC, the area under the ROC curve; BAR, blood urea nitrogen-to-albumin ratio; BUN, blood urea nitrogen; ALB, albumin; GAP, gender-age-physiology.

The diagnostic efficacy of the BAR, BUN, 1/ALB, and GAP index for 1-year all-cause mortality in IPF patients is shown in Figure 3 and Table 2. The area under the ROC curve (AUC) for the BAR to predict 1-year all-cause mortality in IPF was 0.671 (95% CI 0.568–0.774, *p* = 0.002), BUN was 0.637 (95% CI 0.531–0.743, *p* = 0.012), 1/ALB was 0.644 (95% CI 0.539–0.750, *p* = 0.008), and the GAP index was 0.710 (95% CI 0.608–0.811, *p* < 0.001). The DeLong test showed that the diagnostic efficacies of the BAR and the GAP index for 1-year all-cause mortality in IPF patients were not significantly different (*p* = 0.511). In addition, the threshold value of the BAR for predicting 1-year all-cause mortality in IPF patients was 0.12 mmol/g, and the sensitivity and specificity were 0.80 and 0.51, respectively.

**Table 2:** Comparison of the ROC curve analysis of the BAR, BUN, 1/ALB, and GAP index for predicting 1-year all-cause of mortality in IPF.

Parameters	AUC	95% CI	Cut-off value	Sensitivity	Specificity	Youden’s index	*p*- value
BAR	0.671	0.568–0.774	0.12	0.80	0.51	0.31	0.002
BUN	0.637	0.531–0.743	4.45	0.77	0.45	0.23	0.012
1/ALB	0.644	0.539–0.750	0.03	0.57	0.70	0.27	0.008
GAP index	0.710	0.608–0.811	3.50	0.63	0.72	0.35	0.000

ROC, receiver operating characteristic; AUC, the area under the ROC curve; 95% CI, 95% confidence interval; BAR, blood urea nitrogen-to-albumin ratio; BUN, blood urea nitrogen; ALB, albumin; GAP, gender-age-physiology.

**Figure 4:**
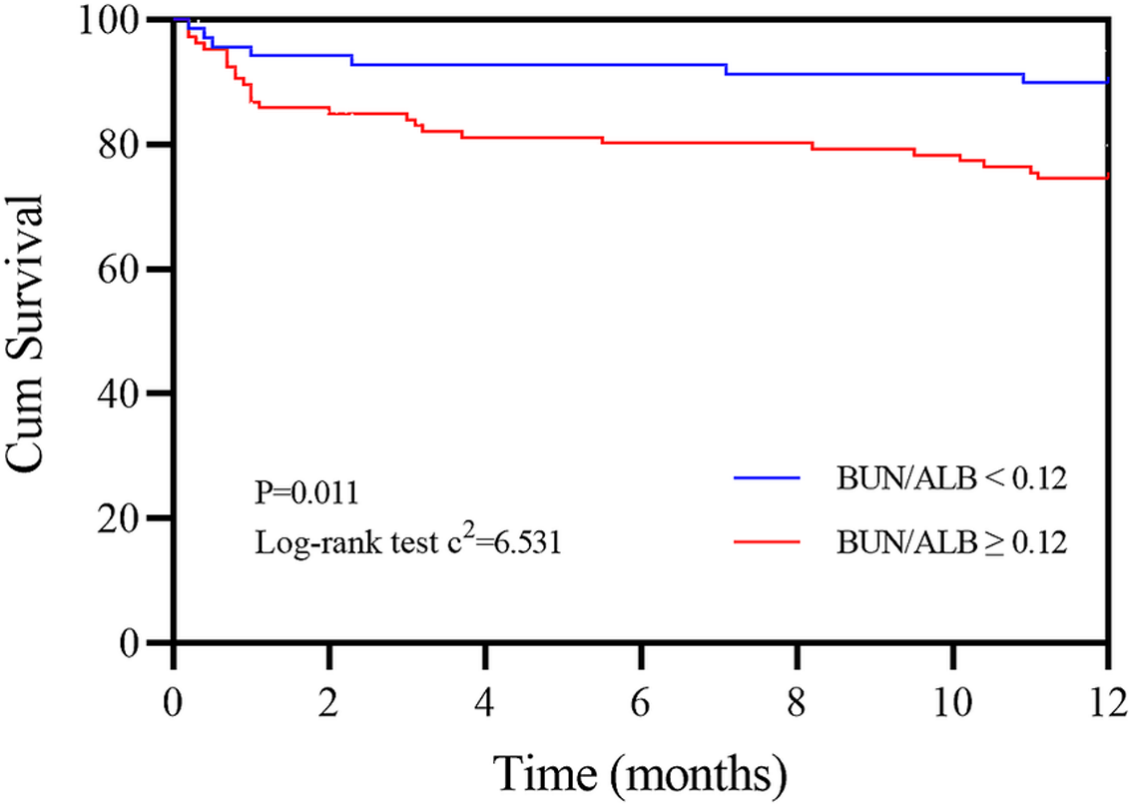
Kaplan–Meier survival curves of IPF patients according to the cut-off value of the BAR. The red line refers to BAR values ≥0.12 mmol/g, and the blue line refers to BAR values <0.12 mmol/g. BAR, blood urea nitrogen-to-albumin ratio.

The study population was further divided into the groups of BAR≥0.12 and < 0.12 according to the cut-off value of the BAR, and the basic clinical information of the two groups is shown in Supplementary Table S1. Compared to the BAR<0.12 group, the 1-year all-cause mortality rate of IPF patients in the BAR≥0.12 group was significantly increased (26.1% vs. 10.1%, *p* = 0.011). The Kaplan–Meier survival curves demonstrated that the 1-year cumulative survival rate of IPF patients was significantly decreased when the value of the BAR was ≥0.12 mmol/g (log-rank test c^2^ = 6.531, *p* = 0.011, Figure 4).

### Cox proportional hazards analysis for 1-year all-cause mortality in IPF patients

The factors affecting 1-year all-cause mortality in IPF patients were detected using univariate and multivariate Cox proportional hazards regression models, and the results are presented in Table 3. The univariate Cox regression analysis showed that BAR ≥0.12 was a significant factor for 1-year all-cause mortality in IPF patients (HR = 2.804, 95% CI 1.224–6.420, *p* = 0.015). The multivariate Cox regression analysis further showed that BAR ≥0.12 remained an independent predictor of high death risk in IPF patients (HR = 2.778, 95% CI 1.020–7.563, *p* = 0.046). Moreover, the coexistence of coronary heart disease, use of antifibrotic medication, pH, OI, FVC, and DL_CO_ were all significantly correlated with 1-year all-cause mortality in IPF patients (*p* < 0.05).

**Table 3:** Influence of the BAR on 1-year all-cause mortality in IPF patients by univariate and multivariate Cox proportional hazards analysis.

Variable	Univariate analysis		Multivariate analysis	
	HR (95%CI)	*p*- value	HR (95% CI)	*p*- value
Age (yr.)	1.002 (0.969–1.036)	0.924	0.971 (0.934–1.010)	0.141
Male subjects (female vs. male)	2.288 (0.888–5.898)	0.087	3.718 (0.991–13.941)	0.052
BMI (kg/m^2^)	0.835 (0.735–0.949)	0.006	1.004 (0.871–1.157)	0.958
Smoking index (pack-year)	1.000 (0.999–1.001)	0.886	0.990 (0.969–1.011)	0.337
Hypertension (yes vs. no)	1.190 (0.592–2.392)	0.624		
Diabetes (yes vs. no)	0.793 (0.329–1.910)	0.605		
Coronary heart disease (yes vs. no)	3.066 (1.391–6.758)	0.005	5.167 (1.874–14.246)	0.002
Antifibrotic treatments (yes vs. no)	0.174 (0.085–0.359)	0.000	0.199 (0.071–0.560)	0.002
pH (≤7.45 vs. >7.45)	3.130 (1.589–6.166)	0.001	3.604 (1.335–9.729)	0.011
PaCO_2_ (mmHg)	0.940 (0.873–1.013)	0.105		
OI (mmHg)	0.988 (0.985–0.992)	0.000	0.987 (0.982–0.992)	0.000
FVC (% predicted)	0.952 (0.934–0.971)	0.000	0.952 (0.929–0.975)	0.000
DL_CO_ (% predicted)	0.970 (0.952–0.989)	0.002	0.975 (0.952–0.999)	0.043
DBIL (μmol/L)	1.007 (0.972–1.043)	0.709		
IBIL (μmol/L)	1.040 (0.972–1.112)	0.255		
ALT (IU/L)	1.016 (1.000–1.031)	0.046	0.976 (0.947–1.005)	0.100
AST (IU/L)	1.034 (1.015–1.054)	0.001	1.033 (1.000–1.066)	0.050
Globulin (g/L)	1.032 (0.978–1.088)	0.252		
Creatinine (μmol/L)	1.016 (0.997–1.035)	0.095		
eGFR (ml/min/1.73m^2^)	1.003 (0.981–1.025)	0.822		
Cystatin C (mg/L)	6.933 (2.181–22.041)	0.001	2.966 (0.539–16.323)	0.211
BAR (< 0.12 vs. ≥0.12 mmol/g)	2.804 (1.224–6.420)	0.015	2.778 (1.020–7.563)	0.046
Leukocyte count (× 10^9^ /L)	1.098 (1.031–1.170)	0.004	0.983 (0.882–1.095)	0.756
HGB (g/L)	0.999 (0.982–1.017)	0.953		
Platelet count (× 10^9^ /L)	1.002 (0.998–1.007)	0.350		

Data are expressed as mean ± standard deviation or median (interquartile range) or percentage. BAR, blood urea nitrogen-to-albumin ratio; HR, hazard ratio; 95% CI, 95% confidence interval; BMI, body mass index; PaCO_2,_ partial pressure of carbon dioxide in arterial blood; OI, oxygenation index; FVC, forced vital capacity; DL_CO_, diffusion capacity of carbon monoxide; DBIL, direct bilirubin; IBIL, indirect bilirubin; ALT, alanine aminotransferase; AST, aspartate aminotransferase; eGFR, estimated glomerular filtration rate; HGB, hemoglobin.

**Figure 5:**
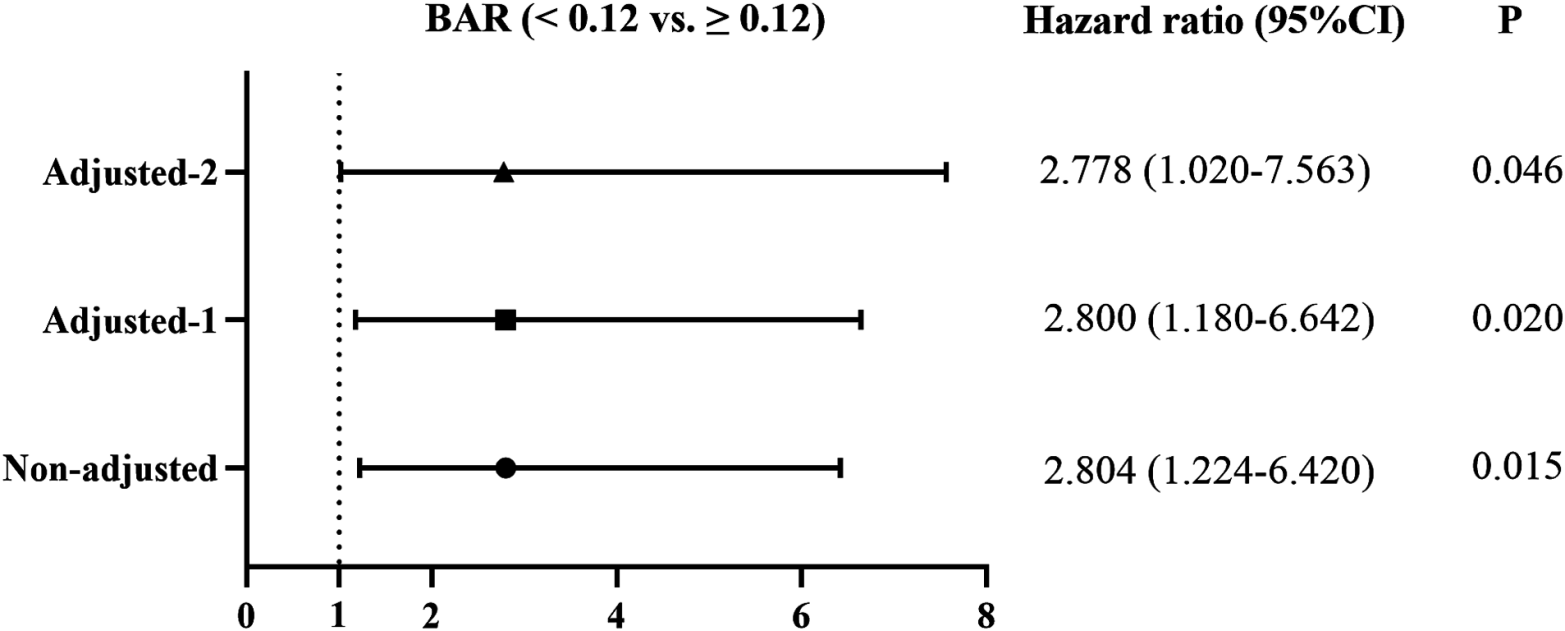
Effect of the BAR on 1-year all-cause mortality of IPF patients using the Cox proportional hazards regression models. Non-adjusted: Univariate Cox proportional hazards analysis; Adjusted-1: Adjusted for age, sex, BMI, smoking index, coexistence of coronary heart disease, and use of antifibrotic treatment. Adjusted-2: Adjusted for age, sex, BMI, smoking index, coexistence with coronary heart disease, use of antifibrotic treatment, pH, OI, FVC, DL_CO_, ALT, AST, cystatin C, and leukocyte count. BAR, blood urea nitrogen-to-albumin ratio; BMI, body mass index; OI, oxygenation index; FVC, forced vital capacity; DL_CO_, diffusion capacity of carbon monoxide; ALT, alanine aminotransferase; AST, aspartate aminotransferase.

Three models were further established to explore the correlations between the BAR and 1-year all-cause mortality in IPF using the Cox proportional hazards regression, and the results are presented as a forest plot (Figure 5). The unadjusted HR value of the BAR for predicting 1-year all-cause mortality in IPF patients was 2.804 (95% CI 1.224–6.420, *p* = 0.015). After adjusting for demographic characteristics (age, sex, BMI, smoking index, coexistence with coronary heart disease, and use of antifibrotic medication), the HR value was 2.800 (95% CI 1.180–6.642, *p* = 0.020). The HR value of BAR remained significant when all demographic characteristics and clinical variables were included in the Cox proportional hazards regression model (HR = 2.778, 95% CI 1.020–7.563, *p* = 0.046).

## Discussion

IPF is a chronic, progressive, lethal, and age-associated interstitial lung disease with a poor prognosis and limited treatment options (14). It has been reported that the median survival time for IPF patients aged 65 years or older in the United States was only 3.8 years (15). The median mortality of IPF patients in Europe was 3.75 per 100,000 and 1.50 per 100,000 for men and women, respectively, based on the World Health Organization mortality database (16). The primary causes of death in IPF patients are the progression of lung disease and its coexistence with other diseases, including ischemic heart disease, lung cancer, pneumonia, pulmonary embolism, and COPD (2). Numerous biomarkers can be used to early identify IPF patients with a high death risk, including demographic data (age, gender, and smoking status), clinical parameters (dyspnea score and lung function), specific radiological features from high-resolution CT images, cytokines in bronchoalveolar lavage fluid, and lung tissue pathology (17). However, these indicators are relatively expensive to obtain or do not accurately predict the prognosis of IPF. Therefore, it is very important to explore accessible and reliable prognostic biomarkers for IPF.

BUN is a main product of protein metabolism and an important indicator of renal function, metabolic status, and inflammation degree (18, 19). Serum albumin is mainly synthesized by the liver, and its level is correlated with liver function, nutrition status, and inflammation (20, 21), while the BAR is a composite indicator consisting of BUN and albumin and has been used as a prognostic biomarker for several inflammatory diseases owing to its association with inflammation and malnutrition. For example, it has been demonstrated that the BAR can be used as a prognostic indicator for hospital-acquired pneumonia (22), acute pulmonary embolism (23), gastrointestinal bleeding (24), and chronic heart failure (25). It is well known that IPF is a chronic, inflammatory, progressive, and wasting disease (26), and usually associated with high levels of inflammation, insufficient protein intake, and malnutrition (27, 28). However, it is unknown whether the BAR can be used as a prognostic indicator for IPF patients.

Our present study has found that the BAR was significantly increased in the non-survivor patients with IPF and positively correlated with the well-known IPF prognostic biomarkers of FVC and DL_CO_ (29), suggesting that elevated BAR may indicate a poor outcome in patients with IPF. However, the pathophysiological mechanisms of BAR to predict the prognosis of IPF patients are unclear, which may be explained by the roles of BUN and albumin in the disease. IPF patients usually experience both pulmonary and systemic inflammatory responses, along with a state of malnutrition, which contribute to an increase in BUN levels and a decline in albumin. Our present study also demonstrated that BUN was increased and albumin was decreased in IPF patients from the non-survivor group, which resulted in the elevation of BAR values in the non-survivors.

The diagnostic efficacy of the BAR in predicting 1-year all-cause mortality in IPF patients was analyzed using ROC curves. Although the AUC of the BAR for predicting 1-year all-cause mortality in IPF patients was smaller than that of the well-known predictor GAP index, the difference was not significant using the DeLong test, suggesting that the BAR is another promising prognostic biomarker for IPF patients. According to the cut-off value of the BAR, IPF mortality was also significantly increased when the BAR value was ≥0.12. The Kaplan–Meier survival curves further confirmed a lower cumulative survival rate in the elevated BAR group, indicating a potentially unfavorable prognosis with higher BAR levels.

The predictive effect of the BAR for 1-year all-cause mortality in IPF may be influenced by several confounding factors; thus, multivariate Cox regression models were applied to account for these variables. Our results have demonstrated that the unadjusted HR of the BAR for 1-year all-cause mortality in IPF was 2.804, which was reduced to 2.800 after adjusting for demographic characteristics (age, sex, BMI, smoking index, use of antifibrotic medication, and coexistence of coronary heart disease) and further to 2.778 after controlling for demographic characteristics and clinical variables. These results demonstrated that the BAR was an independent serum biomarker for 1-year all-cause mortality in IPF patients.

In addition, we found that the coexistence of coronary heart disease was a high-risk factor for IPF death, which may be related to the increased oxygen consumption by coronary heart disease (30, 31). It is well known that antifibrotic medication (pirfenidone or nintedanib) can significantly improve the outcomes of patients with IPF (32), and our present study also confirmed that antifibrotic therapy was a favorable prognostic factor for IPF patients. Consistent with previous findings (29, 33), our present study further showed that OI, FVC, and DL_CO_ were the independent prognostic biomarkers for 1-year all-cause mortality of IPF patients. However, the multivariate Cox regression model showed that BMI was not an independent biomarker for 1-year all-cause mortality in IPF, which may be related to the fact that BMI is not the best parameter for nutritional status. Further studies are needed to explore whether other parameters for nutritional status, such as weight loss and creatinine height index, can be used as independent prognostic biomarkers for IPF.

This study has certain limitations that should be considered. First, it was a retrospective observational single-center study. We did not analyze the effect of therapeutic interventions on the BAR values in IPF patients, including long-term treatments after discharge from the hospital. Second, the degree of pulmonary fibrosis in HRCT can be semi-quantitatively analyzed by experienced radiologists or quantitatively evaluated using specialized software. Due to the instability of semi-quantitative results and the lack of specialized software, the degree of pulmonary fibrosis in IPF patients was not assessed in our present study. In the future, some new techniques or software can be used to quantify pulmonary fibrosis on HRCT and explore their correlations with the BAR. Third, the specific causes of death in IPF patients cannot be accurately obtained due to the retrospective follow-up in our study, and thus, it was not possible to illustrate the relationship between the BAR and various causes of death in IPF. Fourth, the patients participating in this study were all inpatients due to the acute exacerbation of IPF, which may not represent all types of IPF patients, such as patients with a stable status or early stage. Therefore, the results of this study need to be further verified by large-scale multicenter prospective studies in the future.

In conclusion, elevated BAR levels upon admission may be an independent risk factor for 1-year all-cause mortality in patients with IPF. The BAR is a cost-effective and readily accessible parameter that can be used clinically to predict the prognosis of IPF.

## Data Availability

The raw data supporting the conclusions of this article will be made available by the authors, without undue reservation.
