# Supplementary material for: Blood urea nitrogen-to-albumin ratio as a new prognostic indicator of 1-year all-cause mortality in patients with IPF
Source: Front Med (Lausanne). 2025 Jan 6;11:1497530. doi: 10.3389/fmed.2024.1497530 (PMC11743257; doi:10.3389/fmed.2024.1497530)
Supplement: Supplementary file 1 [file Table_1.docx]

**Table S1** Baseline characteristics of the study population stratified by BAR

| Characteristic | BAR < 0.12 (n=69) | BAR ≥ 0.12 (n=107) | P-value |
| --- | --- | --- | --- |
| Age (yr.) | 66.42 ± 9.05 | 67.43 ± 10.53 | 0.513 |
| Male (%) | 59.4 | 82.2 | 0.001 |
| BMI (kg/m^2^) | 23.39 ± 2.55 | 23.05 ± 2.84 | 0.419 |
| Smoking index (pack-year) | 0.00 (0.00-27.50) | 10.00 (0.00-30.00) | 0.152 |
| Smoking status |  |  |  |
| Never (%) | 58 | 43 |  |
| Former (%) | 8.7 | 15.9 |  |
| Current (%) | 33.3 | 41.1 |  |
| Comorbidity |  |  |  |
| Hypertension (%) | 30.4 | 30.8 | 0.594 |
| Diabetes (%) | 15.9 | 24.3 | 0.184 |
| Coronary heart disease (%) | 7.2 | 12.1 | 0.295 |
| Anti-fibrotic medication (Pirfenidone or nintedanib, %) | 88.4 | 89.7 | 0.078 |
| pH | 7.43 ± 0.03 | 7.43 ± 0.04 | 0.776 |
| PaCO_2_ (mmHg) | 37.69 ± 4.38 | 37.88 ± 6.71 | 0.838 |
| PaO_2_ (mmHg) | 75.64 ± 12.93 | 73.37 ± 13.03 | 0.261 |
| OI (mmHg) | 345.76 ± 74.14 | 330.10 ± 82.26 | 0.202 |
| FVC (% predicted) | 77.88 ± 18.37 | 71.97 ± 18.10 | 0.032 |
| DL_CO_ (% predicted) | 74.27 ± 25.11 | 67.56 ± 20.38 | 0.054 |
| GAP index | 3.00 (2.00-3.00) | 3.00 (2.00-4.00) | 0.009 |
| DBIL (μmol/L) | 3.20 (2.38-3.85) | 3.00 (2.25-4.57) | 0.827 |
| IBIL (μmol/L) | 10.08 (7.52-13.07) | 9.10 (6.10-12.16) | 0.101 |
| ALT (IU/L) | 14.00 (11.00-22.50) | 17.00 (11.00-26.00) | 0.178 |
| AST (IU/L) | 21.00 (18.00-23.50) | 21.00 (18.00-28.00) | 0.515 |
| Albumin (g/L) | 37.61 ± 4.16 | 35.12 ± 4.82 | 0.001 |
| Globulin (g/L) | 29.80 (25.55-32.60) | 28.50 (25.10-32.90) | 0.549 |
| BUN (mmol/L) | 3.50 ± 0.73 | 6.17 ± 1.63 | 0.000 |
| Creatinine (μmol/L) | 52.59 (45.09-59.24) | 59.69 (50.95-70.24) | 0.000 |
| eGFR (ml/min/1.73m^2^) | 102.60 ± 11.96 | 98.67 ± 16.71 | 0.092 |
| Cystatin C (mg/L) | 0.94 (0.85-1.05) | 1.07 (0.92-1.24) | 0.000 |
| Leukocyte count (×10^9^/L) | 7.05 (6.08-8.28) | 6.78 (5.70-10.00) | 0.979 |
| HGB (g/L) | 138.70 ± 17.43 | 136.74 ± 20.81 | 0.518 |
| Platelet count (×10^9^/L) | 202.00 (170.50-260.50) | 190.00 (142.00-239.00) | 0.033 |

Data are expressed as mean ± standard deviation or median (interquartile range) or percentage.

BAR, blood urea nitrogen-to-albumin ratio; BMI, body mass index; PaCO_2_, partial pressure of carbon dioxide in arterial blood; PaO_2_, partial pressure of oxygen in arterial blood; OI, oxygenation index; FVC, forced vital capacity; DL_CO_, diffusion capacity of carbon monoxide; GAP, gender-age-physiology; DBIL, direct bilirubin; IBIL, indirect bilirubin; ALT, alanine aminotransferase; AST, aspartate aminotransferase; BUN, blood urea nitrogen; eGFR, estimated glomerular filtration rate; HGB, hemoglobin.
